# “I came to escort someone”: Men’s experiences of antenatal care services in urban Ghana—a qualitative study

**DOI:** 10.1186/s12978-021-01152-5

**Published:** 2021-05-26

**Authors:** Gloria Abena Ampim, Astrid Blystad, Albert Kpoor, Haldis Haukanes

**Affiliations:** 1 Department of Health Promotion and Development, University of Bergen, Bergen, Norway; 2 Department of Global Public Health and Primary Care, University of Bergen, Bergen, Norway; 3 Department of Sociology, University of Ghana, Accra, Ghana

**Keywords:** Men’s involvement, Antenatal care, Space and gender, Ghana

## Abstract

**Background:**

Male involvement in maternal healthcare has been widely recognized as essential for positive health outcomes for expectant mothers and their unborn babies. However, few studies have explored men’s experiences of maternal health services. The purpose of this paper is to explore men’s involvement in antenatal care in urban Ghana and to discuss how men navigate their roles in a space that has been constructed as feminine. The study draws upon theories of space, place, and gender.

**Methods:**

A qualitative exploratory study using semistructured interviews, focus group discussion, and observation was conducted in Accra, Ghana. Expectant fathers and health workers were interviewed, and observation was conducted at a selected public hospital in Accra.

**Results:**

The findings suggest that the few men who attend antenatal care with their expecting partners become involved to a limited extent in the clinic’s activities. Beyond a few who take an active role, most men stay on the outskirts of the hospital grounds and rarely participate in consultations with their partner and midwife. Men still view their presence as necessary to acquire knowledge and as sources of emotional, financial, and physical support for their partners. On the health workers’ side, the study found no clear agenda for engaging men at the clinic, and nurses/midwives felt there was a lack of staff who could engage more directly with the men.

**Conclusion:**

The study indicates that most expecting fathers feel too shy and uncomfortable to locate themselves in the female space that makes up antenatal care/maternity wards. Health workers do not feel they have the necessary resources to involve men fruitfully. Thus, men do not engage in the activity as hoped but rather remain on the outskirts of the maternity clinic. However, if men continue to negotiate their involvement at the clinic and become more assertive in their roles, the maternity clinic as a female space could, with time, be transformed into a space in which both expecting mothers and fathers can actively participate and be engaged to the benefit of all.

## Plain Language Summary

This article discusses men’s roles and involvement in health workers’ activities when they accompany their pregnant partners to the maternity clinic. Health workers organize antenatal care services (ANCs) for expectant mothers to receive regular check-ups during pregnancy. Since pregnancy and childbirth are generally viewed as women’s domains, men have not traditionally been expected to attend antenatal care with their partners. However, recent national and global agendas have recognized men’s inclusion in maternal healthcare as central to improving mothers’ and unborn babies’ health. Men are being encouraged to play an active role in supporting their partners during pregnancy, and as part of this role, they are encouraged to attend antenatal care services. In the health facility where this study was conducted, we found that the few men who attended antenatal care most of the time stayed outside the maternity clinic under trees or in other empty spaces around the clinic. They opted to remain in the outside areas because they felt shy sitting among women who were a substantial majority at the clinic. We also found that health workers rarely involved men in ANC activities because of a lack of staff to engage men in separate sessions. Although the fathers attending antenatal care were disappointed that they were not engaged in activities, they still found it necessary to attend to give their partners emotional, physical, and financial support. Without the consideration of how ANC activities are structured and the appropriate resources for health workers, men’s active participation in ANCs will remain minimal.

## Background to the study

In sub-Saharan Africa, men have, to a large extent, been viewed as the leading decision-makers in the household responsible for the financial resources of the family [1–4]. Men’s ideal roles as leaders and providers in the household have implications for women’s access to quality healthcare during pregnancy and childbirth. When and where to seek healthcare and how much to spend on healthcare, as well as decisions about the number of children in a family largely depend on male partners [4–9]. Positive health outcomes for women and children have been associated with male involvement in both developed and developing countries [10–12]. Consequently, male involvement in maternal and infant healthcare has been encouraged to improve women’s and children’s health and promote gender equality in reproductive health responsibilities [13–16].

Research has shown that male involvement is significant in women’s use of maternal health services [13, 17–19]. In addition to providing material resources to facilitate attendance, men can use their influence to demand respectful care and act as patient advocates [20–22]. However, a number of factors, such as the fear of having to be HIV tested, long waiting hours, the attitude of health workers toward men and the idea that pregnancy is a woman’s responsibility, have been found to prevent men from visiting maternity clinics with their partners [7, 23–30]. Among the limited research conducted on the experiences of men who attend antenatal care (ANC), a study from Rwanda indicates that even when men accompanied their partners for antenatal services, midwives prevented them from participating in private consultations to protect their professional domains and maintain the maternity clinic as a space for women [31]. A study from Malawi on men’s labor and birth experiences found that men experienced increased knowledge but felt fearful, embarrassed, and helpless when witnessing their partners in labor [32].

Studies on male involvement in reproductive health in Ghana have primarily focused on family planning [5, 9, 33, 34]. Other research in the field has discussed factors that prevent men from attending maternal healthcare clinics and has identified expected gender norms, lack of time and low formal educational status as the causes of low male attendance [2, 4, 8, 35–42]. Hence, the experiences of men who try to participate in maternity care services have remained undocumented in the Ghanaian research-based literature. This article focuses on men who attend antenatal care (ANC) with their partners, and it attempts to enhance the knowledge about and understanding of expectant fathers’ experiences of the service. Given the premise that men’s involvement in ANC strengthens reproductive health, knowledge about their experiences at clinics is important to indicate what may be productive or achievable. This study presents the varying forms of expectant fathers’ involvement in ANC and how the organization of the activities and space at the maternity clinic shape what fathers do while there. We draw upon theories of space, place and gender to augment the understanding of the material [43].

### Conceptual framework

Following Doreen Massey [43], we understand space to comprise social relations, while a place is where these relations are performed, constructed, contested and renewed. The formation and identity of a place, its social structure, political character and local culture are all products of interactions [[43], p. 120]. Both spaces and places are therefore formulated in terms of social practices. Gender is influential in defining the kind of relations that are played out in particular places and in shaping the way that men and women relate in a place. Massey [43] noted that the dominant image of a place and space will often be contested and subject to gradual transformation.

The place under discussion in this context is a maternity clinic where expectant mothers gather as a group led by health workers. The maternity clinic can be described as an example of a “third place”. Third place has been defined as “physical locations outside the home (first place), or workplace (second place) that facilitate social interaction, community building and social support” [[44], p. 1] [45]. The maternity clinic is a physical location where women and their caregivers share reproductive health information and a place where women form social relationships with other expectant mothers. In this way, the clinic supports and sustains women’s social life during pregnancy.

The continuous interaction of pregnant women and their caregivers at the maternity clinic, discussing reproductive health matters, has constructed the maternity clinic as a female space where masculine presence and domination have been rather peripheral. However, when men visit the maternity clinic, it is expected that they share the same space with their partners, a space where their defined hegemonic roles [46] as heads of the household are of little or no significance. In this regard, we seek to discuss how the maternity clinic as a physical place and socially constructed space is being potentially reshaped to include men and how men negotiate their authority and masculinity within this space.

## Methods

### Study design

This article forms part of a broader study that the authors conducted between June 2017 and May 2019 to explore the nexus of male involvement in maternal healthcare and gender relations in Ghana. The study used an exploratory qualitative research approach to present detailed descriptions of people’s own understanding of events and experiences [47]. Semistructured interviews, focus group discussions (FGDs) and observations were used to gather in-depth knowledge about how activities are organized at the maternity clinic of a key government hospital in Accra.

### Study setting

The study was conducted in Accra, Ghana’s capital. All participants were recruited through one of the fully government-operated hospitals in the Accra metropolis. The particular hospital was selected as the main facility in the study, as it is a key government hospital in Accra and provides services through National Health Insurance. Moreover, a government facility was selected because its clients represent people of different social statuses in Ghana, unlike private facilities that are likely to have wealthier clients.

### Participants

Participants contributing to the data illustrated in this article are expectant fathers, midwives and community health nurses.^1^ Purposive sampling was used to recruit men and health workers. The recruitment proceeded as follows: the first author (GAA) started out by observing expectant mothers and fathers as they came to the maternity clinic. She also joined the antenatal education sessions and took note of the men present, their level of involvement in activities, their body language, and their interaction with service providers and other men and women. She then approached these men after the educational sessions had ended and informed them about the project individually. Observations and recruitment continued over a seven-month period at the maternity clinic. The inclusion criteria for the men were first-time expectant fathers who were regular attendees at ANC. Because expectant mothers are required to attend at least four antenatal visits, one inclusion criterion of expectant fathers was that they had attended ANC at least twice before the interview. All men were recruited at the hospital and followed up on with phone calls to schedule interviews. Follow-ups presented an opportunity for the researcher to establish rapport with the participants and gather more background information before the actual interview took place. Not all men contacted were able to participate in the interviews; some did not meet the inclusion criteria, while others could not find time to participate. Ten men were interviewed. All except one were first-time expectant fathers. One father had a child from a previous relationship, but this was revealed only after the interview.

### Topic guides

GAA conducted all interviews and the focus group discussion using three different open-ended topic guides. The topic guide for the qualitative interviews with the fathers focused on four main topics: fatherhood and masculine norms within the Ghanaian social context; fathers’ roles during pregnancy and childbirth; the role of the extended family during pregnancy and childbirth; and the experiences of fathers at service points, including the maternity clinic. The second topic guide for midwives focused on perceptions about fatherhood norms, benefits of male involvement in service delivery, ways of engaging men at the clinic, and the potential challenges of male involvement at the clinic. The third topic guide used in a focus group discussion with community health nurses (CHNs) focused on perceptions about fatherhood norms, benefits of male involvement as observed in the community and at the health facility, ways of engaging men in maternal health at the clinic as well as in the community, and the potential challenges of securing male engagement at the community level and at the clinic. Because the instruments were open-ended, GAA could adjust the questioning to suit the individual participants’ situation. She also engaged in a continuous review and rephrasing of the questions to ensure that they were clear and comprehensible to the research participants.

### Data analysis

The data were analyzed using Braun & Clarke’s [48] approach to thematic analysis. Data analysis began with the writing of an analytic memo, which was updated periodically during fieldwork. Key concepts that participants mentioned were recorded in an analytic memo and later used to generate codes. Soon after gathering the data, all the tape-recorded audio was transcribed and anonymized to protect the identity of the study participants. Upon transcription, a few interviews were printed out and coded on paper to develop a coding manual. All transcripts were later transferred into QSR NVivo software, where more codes were generated. Themes were generated from the codes, and some themes were merged with other themes upon consultation with the research team. Themes were continuously refined by the coauthors.

### Ethics

The study is guided by the protocols of the Norwegian Institute for Data Protection (53,570/3/ASF). Ethical approval was granted by the University of Ghana, College of Health Sciences (CHS-Et/M.6–P1.12/2017–2018). Permission was also sought from the administration and the maternity clinic of the hospital before the study began. Written or oral informed consent was obtained from all participants after thoroughly explaining the purpose of the study [49]. Moreover, interviews and discussions were recorded only upon the acceptance of the participants. The participants were informed that they could withdraw their consent at any point during the session without any consequences and that information gathered from the study would remain anonymous. All participants were given pseudonyms to enhance anonymity.

## Results

This section presents a description of the maternity clinic, how ANC activities were organized, and where men were located during the clinic’s proceedings. It will then continue with a discussion of the varying forms of male involvement found in the study, including men’s own agendas at the clinic. It ends with a presentation of health workers’ engagement of men at the clinic.

### The maternity clinic and male partners

The maternity clinic was managed by a midwife and offers three fairly distinct services: antenatal care, labor and delivery care, and postnatal care. The *antenatal care* (ANC) division had one gynecologist and approximately four midwives per work shift. The clinic operates between 8 am and 5 pm from Monday to Friday. Mothers began to arrive as early as 6 am to form a queue, sometimes coming in the company of other mothers or partners. By 8 am, when sessions started, the seats in the waiting area where ANC takes place were usually fully occupied by expectant mothers. The sessions commenced with a midwife leading the expectant mothers in the waiting area in a Christian worship session lasting approximately ten minutes. She then led the group in a short exercise session before giving an educational talk on topics such as nutrition, birth preparedness, and signs of labor, among others. More mothers arrived as the educational session proceeded. On average, approximately 200 women attended ANC at the hospital per day.

During the session, mothers talked among themselves and with health workers, asked questions, and shared jokes. When the educational session ended, individual mothers were called upon, and their folders were sorted according to their assigned midwives. At this point, women waited for a one-on-one consultation with their assigned midwives in a separate room. This is a crucial service provided during ANC. Although the waiting area was largely congested during the educational session, the space began to open up as the educational sessions closed and women continuously moved in and out of the clinic. It was common to find expectant mothers moving around in pairs or more to the canteen, washroom, and laboratory. Women who attended the clinic with their partners walked around with them.

Men who accompanied their partners to the clinic were encountered mostly at three different locations. Some sat among the expectant mothers in the waiting area during the educational sessions. From observation, we found only two to three men sitting among the more than 100 women. Others stayed in an open space outside the maternity clinic, while others again were seen under trees on the broader compound of the hospital. Although men may not be present in the waiting area, they were still sometimes called by their partners to join them for the one-on-one consultation. Statistics from the hospital show that between 2016 and 2019, male attendance at the maternity clinic had a ratio of approximately ten mothers to one father. These numbers include men who came for antenatal, labor and delivery care and postnatal care. Labor and delivery care have the most sizeable male attendants, according to health workers. Thus, very few men attended ANC sessions with their partners. From the few men who came for ANC, we found different ways and levels of involvement.

#### Varying involvement of men in antenatal care

The first question that we asked men after greeting them at the maternity clinic was, “What brought you here today?” Most men responded by saying, “I came to escort someone”. This response was also clear in the ways that men and women occupied space at the hospital. While women usually gathered in the waiting area, most men were, in contrast, found waiting under trees and in isolated spaces behind the clinic. Expectant fathers who remained outside during the general session said that they were uncomfortable inside the maternity clinic. One man, Ibrahim, for example, said that he was surprised to see so few men when he attended the clinic with his wife for the first time, and this made him feel uncomfortable. Another expectant father specifically mentioned that he was uncomfortable sitting among the women:

*I would not like to be among the ladies. That place is only meant for the women. So if I come with someone, I would not like to sit *among* the ladies* (Elorm, 30 years old, Artist).

Other expectant fathers decided to make themselves invisible at the clinic, saying it was because they felt shy. When we interviewed one man, Derrick, for the first time, his wife was eight months pregnant. He had attended the ANC since they received a positive pregnancy test. However, sometimes the midwives did not even see him because he was mostly hanging around under the trees surrounding the facility. He did not participate in either the educational or the consultation sessions because he did not see other men doing so. He nonetheless concluded by saying that if he is invited, he will join the educational session. He explained:

*I feel shy. Well, I do not go into the *room* with her unless I am invited. The only time I went inside was when I was called to donate blood. I have not been there since then. If it is allowed, I will go* (Derrick, 32 years old, Truck Driver).

Some men revealed that they were only shy during the first visit and later became more comfortable sitting among the expectant mothers in the waiting area:

*When you come for the first time, you would be shy because you would meet many women, and the reaction from their faces would be like: “Ah, is your wife the only pregnant woman?” Well, fortunately, I do not take notice of such things because I know my purpose there. So I just sit quietly and mind my business* (Joseph, 32 years old, Sales Manager).

Although the seats in the waiting area were mostly fully occupied by expectant mothers, none of the men interviewed mentioned a lack of seating space as the reason they waited outside. Instead, as we have shown above, they related their staying away from the waiting area to experiencing shyness and discomfort. Some expectant fathers were unhappy with a limited level of involvement at the clinic and felt that activities should be tailored to include them, especially in the individual consultations:

*The only time you will see the nurses is when they come to mention the names of those whose cards they have. So when they are calling the names, and you are also following, you know the nurses are rude at times. They ask where you are going and all sorts of questions. In addition, even seeing some of the *men* around, even though most men do not come, at least they should come and ask what we came to do or who we came with and all that. They just move back to the rooms after mentioning the (women’s) names* (Eric, 30 years old, Sales Executive).

Like Eric, some of these men seemed not to be aware that men were allowed to participate in the consultations. Derrick also said he would participate if it were allowed. Ibrahim similarly said:

*We came together, we even went *down* there, but I am currently sitting here because she is going for a scan, and she is the only one expected to be present there. After the scan, the next is the lab and a whole lot before seeing the midwife. That is why I am waiting for her here* (Ibrahim, 32 years old, Trader).

When asked whether he would join his wife in the consultation with the midwife, he answered, “*If I am permitted, why not?*”.

Interaction at the clinic was, for most men, limited to interacting with their partners. There was hardly any communication between the men themselves or between men and women. Most men played on their mobile phones and tablets in their idleness. They explained that it was better to focus on their purpose at the clinic rather than chatting with other men.

The men who kept a distance and did not involve themselves in activities at the clinic were a substantial majority among our study participants. However, there were also a few who were more assertive and actively participated in the consultation with their partners, as shown below:

*As for the consulting room, I always make sure I am there with her because I want to see if everything is in place. So if there is any lab test, I would like to know its result and what to do about it. We went there together yesterday when the time was due for the test they conducted. That was when she (midwife) *told* us what to buy for the child and other things needed for the pregnancy* (Eddie, 28 years old, Self-employed).

Eddie usually joined his partner for the educational session and would after that drop out and wait outside until it was time for her consultation with her assigned midwife when he would be called to join in. Charles was another man who said that he regularly participated in the consultation with the midwife. When observed at the clinic, Charles appeared to be in control and aware of his rights and privileges, leading his wife through the various proceedings, carrying her folder and handbag while she followed behind. Men such as Derrick became more involved in activities toward the end of pregnancy. His wife developed complications in the third trimester, which required that she attended frequent check-ups. Upon the request of his wife’s assigned midwife, Derrick joined the consultations when his wife’s due date was drawing closer.

#### Men’s agenda at the maternity clinic

Irrespective of the varying forms of involvement at the clinic, expectant fathers seemed to have their agenda for attending ANC with their partners. Observations showed that most men at the clinic made payments on behalf of their partners, bought them food, and carried their handbags and folders. One expectant father, Eric, for example, was observed at the clinic sitting in an empty space, holding his partner’s handbag and folder. Although Eric was disappointed that health workers did not involve men in the ANC activities, he found a way to make himself useful. Our study participants also shared that they performed some roles in the form of seeking knowledge and providing emotional and physical support for their partners at the clinic.

*I always want to come here to know more about pregnancy, so I do not take anything for granted. Sometimes, the woman may complain of a headache, and you would not know what that means, but when you come here, and they teach, you would know how to treat such things. It helps me to take good care of her. Sometimes she forgets the things they teach there as well, so when I go there, I take notes like a student so that I do not forget the lessons *(Joseph, 32 years old, Sales Manager).

The quote above indicates two motivational elements of men attending ANC: to acquire knowledge and remind their partners of information relayed at the clinic. Some expectant fathers understood antenatal visits as an extension of their role as the head and protector of their family:

*This is her first time she has been pregnant, and her family members are not here. So I am supposed to support and help her out during this time. I have some questions to ask the midwife. She alone will not *be* able to ask all those questions, you know. How will she do all that because she is a young girl and does not know anything about pregnancy* (Elorm, 32 years old, Artist).

Men also claimed that spousal love and affection were a key motivation for their involvement in ANC with their partners. When asked why he continued to attend ANC with his partner, Charles answered, “The woman, she is good”. Similarly, men emphasized that attending ANC was an expression of love for their partners. They explained that experiencing the process of pregnancy with their partners will promote respect for women, as indicated in the two quotes below:

*I am happy that I came here with her because she knows that I support her. Just staying away from work for a day for her will not affect anything. Just knowing that I support her in the pregnancy gives me joy as well* (Martin, 38 years old, Mechanic).

*It is good for every man to go through that process to have some respect for every woman they see. Some men do not respect women. All they care about is, hey, after all, she is just my girlfriend. They do not see them as their fellow human beings and treat them as such. I believe after they go through this experience, their respect and care for women would increase. So I think when you are always there with her throughout the process, there would be a change of mindset on how to treat women. It has truly changed my mind and mentality about women* (Joseph, 32 years old, Sales Manager).

### Health workers engagement of men at ANC

In general, health workers seemed happy to see men at the clinic, although they had divergent opinions on including men in the procedures. Apart from giving preferential treatment to women whose partners attended ANC, health workers had no clear agenda about facilitating male involvement in maternity services. Irrespective of what midwives viewed as the appropriate way to involve men in ANC, their primary concern was how male attendance demanded an increase in their already substantial workload. They held that it would be more convenient for health workers if ANC focused mainly or only on women.

#### Jumping the queue

Health workers mentioned that there is a recommendation to motivate men who attend ANC by allowing their partners to move more quickly up the line.^2^ This principle is justified by the idea that men have to go to work as providers for their families, and therefore, their partners should be allowed to move more quickly up the queue so the men can go back to work. All health workers who participated in this study mentioned this incentive, summarized in the excerpt below:

*When you come with your wife, we give you priority. We see you first. Because among the lot, approximately 200, 300, we have about five men. We treat you as a special guest for that day. That is what we have been doing. Even when I am walking around, and I see a man sitting, I ask the wife, which room do you go to? Then, I tell the midwife in that consulting room, do not forget there is a man there. See that person first* (Naana, Midwife).

However, this incentive and practice were not something we came across in the interviews with the men or in the observation at the clinic. Only one of our study participants, Charles, said that he “helped his wife to jump the queue”. During the follow-up interview after birth, while Charles and his wife were expecting their second child, he still attended ANC to help his wife move more quickly up the line. Interviews and observations showed that very few men at the clinic knew of the incentive to give queue privileges to women who were joined by their partners to ANC. One reason for this might be that health workers in practice seemed to keep silent about this privilege and incentive. Naana, one midwife, explained that expectant mothers waiting for ANC regularly engaged in quarrels about issues relating to the queue and about people they suspected of cheating. Consequently, health workers gave this preferential treatment silently, and only men and women who were already aware of the privilege were able to take advantage of it.

#### Challenges of male involvement

Health workers shared different views on how men should be involved in the activities at the clinic and the challenges involved. Community health nurses talked about inadequate physical space for men at the clinic, while midwives talked about whether men’s inclusion should be prioritized:

*The women at antenatal care are usually very plenty. Sometimes they (men) enter there, and they see plenty of women there, and they will go back. We do not even have space (physical) for the men. Therefore, we are not making it comfortable for men to involve themselves* (CHN, FGD).

CHN’s views on sitting/waiting space at ANC seemed to support men’s concern that sitting among women was uncomfortable. Naana, as shown above, argued that men who accompanied their partners should be treated as unique and served quickly. Nevertheless, she believed that men’s presence should not be a priority unless the condition of a pregnant woman demanded her partner’s participation in ANC services. She gave the following example:

*If your husband is not coming to postnatal with you or the antenatal, we are not bothered. We are not bothered. *We* only need the woman if everything is fine, yeah. Unless she comes and there is a problem. Then, we will call the man* (Naana, Midwife).

Rebecca, another midwife, said that men participating in the consultation would increase their workload. Therefore, she had suggested to the hospital that the morning sessions should be communicated through videos, which would include videos on what men can do to support their partners. Additionally, she emphasized that a separate men’s group would be better than men participating in the private consultation. Nonetheless, this should happen only if and when specific health workers could be assigned to male attendees.

Agnes, another midwife, held a slightly different position and argued that men should participate in the consultations because it would make their work much more manageable:

*As I said before it (male inclusion) is something very good and makes the work simple for us. When you tell them what to do and what not to do, the men remind them (expectant mothers) at home to comply with the instructions. They (men) are always there to check their wives for us* (Agnes, Midwife)*.*

Agnes’ claim here reiterates that health workers support the men’s agenda for attending ANC to acquire knowledge and remind their partners at home of instructions received during the consultation. Unlike Rebecca, Agnes claimed that men’s participation in consultations would neither prolong nor increase the workload. However, separation of the women and men would require additional caregivers and time, which the hospital may not have the capacity to provide. She concluded by saying that it would be possible to organize a separate information session for expectant fathers only when they start coming to the clinic in more significant numbers.

## Discussion

As stated at the onset of the paper, global health research has demonstrated that active male participation in maternal healthcare services improves women’s health outcomes and promotes gender equality in reproductive health [14, 15, 50]. Some literature from sub-Saharan Africa indicates that male partners attending ANC facilitate quality care for women by demanding respectful care and acting as patient advocates at health facilities [20–22]. Nevertheless, male experiences of ANC services have not received much research-based attention. Our study found that only a minority of pregnant women’s partners came to the ANC, and those who did were actively engaged in the activities at the clinic only to a limited extent. While a few men did take an active part even in the individual consultation between their partners and the midwives, asked questions and helped to remember instructions, most men attending this particular ANC seemed to maintain a relatively distanced role.

In an attempt to gain in-depth knowledge and understanding of why men seemed to maintain a distanced role at the maternity clinic, we analyzed the data using the framework of space, place and gender [43]. This framework can elevate our understanding of how a physical place, such as the maternity clinic, can become a site of gender display and explain why men seemed to be marginalized. The identity of the maternity clinic is a product of the activities that have been produced and relations that have been constructed in the location over time [43]. As a physical location where women and health workers (also mainly women) interact and share knowledge on pregnancy and childbirth issues, the place has come to exist as a gendered “female” space.

The clinic also acts as an essential “third place” for expectant mothers. As a physical place located outside the home and the workplace, it promotes social interaction, networking, and support during pregnancy [[44], p. 1]. Antenatal care visits provide physical healthcare and support and sustain women’s social life during pregnancy in the form of sharing information and creating long-standing social relationships and networks, including mother-to-mother support after childbirth. Women may be reluctant to include men to protect the relationships that they forge at ANC and preserve female autonomy over pregnancy and childbirth, as indicated in Ghanaian studies [2, 39].

Furthermore, maternity clinics function as safe spaces where mothers have reportedly preferred to discuss contraceptive use and other matters in their relationships with their peers and health workers without involving their partners, sometimes for fear of intimate partner violence [[2], p. 200]. Spousal violence during pregnancy is quite common, with “prevalence rates of 28–40% for physical, 3–27% for sexual and 25–49% for emotionally intimate partner violence” among pregnant mothers in Africa according to a WHO report [[51] p. 1]. Women who experience violence during pregnancy may be unwilling to attend ANC with their partners. For intentional and unintentional reasons, men have been marginalized in the maternity clinic’s operation and interactions.

Our study suggests that men felt out of place at the maternity clinic. Indeed, at times, they remained almost invisible and physically distanced outside the maternity clinic. An indication of men’s loss of confidence at the maternity clinic is related to their remarks about shyness that triggered their decision to remain hidden or to stay away from the waiting area even when they accompanied their partners. Men conveyed that they were uneasy as a minority in a predominantly female group, a finding that has also been reported in other studies [36]. Hence, they felt more relaxed when they withdrew to a space away from where large numbers of women would often be gathered, either to the outside of the maternity clinic building or to inside areas where the individual consultations took place, where only the man’s wife and the nurse would be present in addition to himself.

Men’s experience of discomfort at the clinic can also be related to their potential loss of autonomy in a space where they feel socially and spatially marginalized. As we have seen, the clinic is organized in a manner where the focus of the health workers, primarily women, is almost solely on the pregnant women. Thus, men, who in a home setting act as heads of the household, find themselves in a space occupied mainly by women. They feel uninvited to participate and have little to contribute to ongoing activities. In some cases, expectant fathers hoped to participate in the private consultation to ask questions that they maintained their partners would not properly ask themselves and to remind their partners of important health instructions. Health workers acknowledged that men who participate in private consultations asked questions relating to their partners’ health. Participating in the private consultation demonstrates men’s efforts to share and contribute to their partners’ maternal health while simultaneously presenting them with the opportunity to feel slightly more in control and reaffirm their masculinity.

Health workers acknowledged that they have some structural constraints that reduce their ability to involve men in ANC activities. Inadequate physical space and staff to organize separate sessions for expectant fathers are a real challenge, as illustrated in our study. This challenge has been documented in other Ghanaian studies, and maternity care services have been criticized as not designed to include men [8]. As part of the Ghana Ministry of Health’s gender policy, health workers are encouraged to engage men to harness their support for appropriate decisions regarding women’s reproductive health [52]. The policy also indicates that health workers should be supported with gender-sensitive training in preservice and in-service education to deliver gender-responsive health services [[52], p. 27]. Although undocumented, health workers claimed that there is a recommendation of giving preferential treatment to women who attend maternal health services accompanied by their partners to motivate men. The reasoning behind this incentive has been that men are breadwinners and therefore need to leave the maternity clinic quickly to go to work.

As articulated by some studies, a significant problem with this incentive is that women find the principle unfair to those who come to the clinic unaccompanied [2, 53]. Health workers in our study have also mentioned that they remain largely silent about giving preferential treatment to women who are accompanied by their partners to avoid conflict among expectant mothers in the waiting area. Moreover, this incentive may give the impression that the male figure is more important than the female figure at the maternity clinic and may reinforce gender discrimination. If such a recommendation were effectively implemented, it could encourage men to attend the clinics. However, in the long run, it could function against the promotion of gender equality in reproductive healthcare.

Despite the global and national emphasis on male involvement in maternal healthcare services [52, 54], men in this study assumed a minimal participatory role in ANC visits. They often remained outside the maternity buildings or even in more distant hospital quarters and primarily accompanied their partners to and from the hospital. A few became disappointed when they found that they were not expected or even allowed to take a more active and engaged role at the clinic. In this study, men’s disappointment about the lack of facilitation to participate in consultations is similar to the findings from a Rwandan study where men were also reported to be disappointed that they were not involved beyond attending ANC [31]. Nonetheless, our study’s findings indicate that attendance at the ANC implies a journey where men seemed to gradually become more involved; they become more accustomed to the setting and activities, and in the process, they become less wary of their own presence in a female space.

As Massey [43] has argued, the identity of places and spaces are subject to contestation and change over time. In this line of thinking, could the maternity clinic as a gendered space be transformed to accommodate a male presence with more active participation and inclusion of men? As we have shown, men’s participation is limited and involves engaging with health workers only to a limited extent. Men felt anxious, invisible or out of place in the maternity clinic. However, we have also seen that male attendees strived to make themselves useful in a way that was compatible with their positions as heads of the household. Typical examples of such involvement found among study participants included blood donation, making payments, buying medication, and carrying their partner’s bags and folders. Men also talked about reminding their partners of important health messages and instructions from the clinic. Moreover, our data indicated that men were motivated by love for their partners to become involved in ANC.

In summary, our findings suggest that men have actively initiated their participation in ANC more than health facilities and health workers. The examples of male involvement in ANC presented here substantiate the assertion that men taking joint responsibility for women’s workload during pregnancy fosters a less stressful prenatal experience for expectant mothers [55]. Our research material also emphasizes the claim that men become exposed to new ways of relating to their partners through their involvement in pregnancy-related care, which could promote new fatherhood norms [55]. Perhaps with a strategic focus on the clinic’s spatial setup and including men in the general sessions and private consultations, the maternity clinic could, with time, be transformed into less of a gendered space, i.e., a space that will accommodate both female and male identities.

## Conclusion

This study has focused on understanding men’s experience of ANC services using the conceptual framework of space, place and gender. The findings suggest that there are differing levels of male involvement in maternal healthcare services and that active participation of men is influenced by socially expected gender norms and health facilities’ structural factors. Although men seemed to have their own agenda for attending ANC, they felt uncomfortable in a space that was predominantly occupied by women, and they felt marginalized in the services provided by health workers.

ANC is organized for expectant mothers, and this core purpose should not be neglected out of an eagerness to include male partners. Concurrently, the importance of the presence of men who accompany their partners to ANC for various reasons cannot be overlooked. Active male participation in ANC offers an opportunity for the mobilization of men to reflect on and nurture new fatherhood norms and new ways of relating with their partners. Therefore, more research on men’s experiences in ANC services at other government hospitals in Ghana will facilitate gathering the best evidence for appropriate ways to organize and stimulate gender-transformative norms.

An examination of the capacity-building needs of health facilities involving men in pregnancy and childbirth-related care would be beneficial. Considering the often-limited physical space and the limitation of health workers to engage men in separate quarters at ANC, health facilities should, at the discretion of health workers, assess what is possible within their capacity and resources to accommodate male attendees. Audio-visual materials for broadcasting male activities and responsibilities during pregnancy in maternity clinics’ waiting areas may be further explored. Furthermore, gender-sensitive education in the preservice and in-service training of health workers could be enhanced.

Following the global surge in promoting male involvement in reproductive healthcare to improve women’s and children’s health and advance gender equality in reproduction, it is important to investigate and address the physical and social formation of maternity clinics. Without appropriate spatial arrangements and resources for health facilities and health workers, men attending ANC will not feel adequately involved, as highlighted in this qualitative study**.**

## Data Availability

The datasets used and/or analyzed during this study are available from the corresponding author on reasonable request.

## Abbreviations

ANC: Antenatal care
CHN: Community Health Nurse
FGD: Focus group discussion

## Footnotes

1 As part of the broader study, interviews were also conducted with expectant mothers in Accra, and health workers and expectant parents in rural areas, but these interviews do not form part of the data used for this article.

2 Health workers spoke about this incentive in the form of a policy that is implemented in all government health facilities in Ghana. However, we found no documentation to show that the Ghana Health Services has a policy that permits giving preferential treatment to women who attend the ANC with their partners. Another study by Ganle et al. 2016. “If I go with him, I cannot talk with other women,” also mentioned a health facility giving preferential treatment but not as part of a health sector policy.
